# Transarterial chemoembolization for unresectable hepatocellular carcinoma: a comparative study between transradial and transfemoral approaches

**DOI:** 10.3389/fonc.2025.1553939

**Published:** 2025-03-20

**Authors:** Shun-Ting Bo, Jun Zhu, Li-Xiao He, Xiao-Li Zhu

**Affiliations:** ^1^ Department of Interventional Radiology, The Third People's Hospital of Yancheng City, Yancheng, China; ^2^ Department of Interventional Radiology, The First Affiliated Hospital of Soochow University, Suzhou, China; ^3^ Department of Day Care, Binzhou People’s Hospital, Binzhou, China

**Keywords:** radial, femoral, TACE, hepatocellular carcinoma, radiation

## Abstract

**Background:**

While transarterial chemoembolization (TACE) has been performed using both the transfemoral approach (TFA) and the transradial approach (TRA) to date, the relative superiority of these techniques remains uncertain. This study aimed to evaluate the relative clinical efficacy, radiation exposure, and safety associated with TRA- and TFA-based TACE procedures in patients with inoperable hepatocellular carcinoma (HCC).

**Methods:**

This study compared the relative outcomes of consecutive inoperable HCC patients who were treated via TFA- or TRA-based TACE between January 2020 and August 2024.

**Results:**

This retrospective analysis included 83 HCC patients, of whom 41 and 42 respectively underwent TFA- and TRA-based TACE. Both of these approaches were associated with technical success rates of 100%. The mean TACE duration in the TRA group was significantly shorter than that in the TFA group (57.4 ± 23.5 vs. 73.5 ± 23.3 min, P = 0.002), whereas both groups exhibited a similar median fluoroscopy time (14 min vs. 13 min, P = 0.415) and radiation dose (159 Gy.cm^2^ vs. 160 Gy.cm^2^, P = 0.946). Two patients in each group experienced puncture site hematomas (4.9% vs. 4.8%, P = 1.000). While patients in the TFA group required ≥ 20 h of postoperative bed rest, the same was not true for patients in the TRA group.

**Conclusion:**

TRA- and TFA-based TACE are both safe and feasible approaches to treating inoperable HCC patients. Relative to the TFA-based approach, the TRA-based approach entails a significantly shorter arterial compression time and requires less postoperative bed rest.

## Introduction

Transarterial chemoembolization (TACE) is widely used for the management of patients suffering from unresectable hepatocellular carcinoma (HCC) (1–3). TACE and other vascular interventions are often performed via the transfemoral approach (TFA) (4–6). The transradial approach (TRA), however, has recently emerged as an alternative technique used for vascular interventions owing to its association with better patient satisfaction (5, 7, 8).

The TRA, however, is also associated with potential limitations including puncture failures, cannulation difficulties, and the risk of radial artery occlusion (9, 10). The routine implementation of TRA-based treatment strategies has also been hampered by the potential for patients to be exposed to elevated radiation doses, the longer distance from the puncture site, and the more challenging learning curve associated with such procedures (11, 12).

TRA-based approaches are most commonly used in the context of cardiovascular and cerebrovascular interventions at present (5, 10). Relative to the TFA, the TRA has been reported to be associated with a shorter puncture time, lower complication rates, and more rapid recovery postoperatively (5, 13). In patients undergoing elective TACE procedures, the TRA is associated with improved satisfaction and quality of life without any adverse impact on safety or procedure-related parameters (14). While the technical feasibility of TRA-based TACE has been firmly established in individuals with HCC (14), further research is needed to clarify the relative clinical efficacy and safety of TRA- and TFA-based TACE procedures. The radiation exposure levels that these two procedures entail also remain to be established.

This study was developed with the aim of evaluating the relative clinical efficacy, radiation exposure, and safety of TRA- and TFA-based TACE procedures in inoperable HCC patients.

## Materials and methods

### Study design

This was a single-center retrospective analysis. This study included consecutive patients with inoperable HCC who were treated via TRA- or TFA-based TACE between January 2020 and August 2024. Patients were eligible for inclusion if they (a) had been diagnosed with HCC; (b) were not eligible for surgical treatment; and (c) were of Barcelona Clinic Liver Cancer (BCLC) stage A or B. Patients were excluded if they (a) lacked complete clinical data; or (b) had previously undergone surgical resection, TACE, ablation, or other cardiovascular, cerebrovascular, peripheral vascular interventions.

### Diagnosis

Patients in this study were diagnosed with HC through contrast-enhanced computed tomography (CT) and/or magnetic resonance imaging (MRI) based on observed enhancement that was consistent with arterial hypervascularity and venous/delayed phase washout (15). Percutaneous biopsy was performed for those patients without these typical imaging findings.

### TACE

The same group of interventional radiologists performed all TFA- and TRA-based TACE procedures for patients in this study cohort. All procedures were conducted by 2 interventional radiologists with 10 and 15 years of experience.

TFA-based TACE procedures were performed under local anesthesia with fluoroscopic guidance. After puncturing the right femoral artery, a 0.035-inch hydrophilic wire (Terumo) was used to place a 5F RH catheter (Terumo, Tokyo, Japan) in the celiac artery. This catheter was then used to perform angiography to confirm the identification of tumor-supplying blood vessels. This 5F RH catheter was then used to insert a 2.7F micro-catheter (Terumo) which was placed in the tumor-supplying vessels with roadmap guidance. TACE was performed using a combination of 5-fluorouracil (150 mg), epirubicin (50 mg), mitomycin C (10 mg), and lipiodol (10-20 ml). Angiography was then repeated to assess any residual tumor staining. When TACE was complete, the puncture site was manually pressed for approximate 10 minutes, and then the mechanical compression with a pressure bandage for 4 h was used to achieve femoral puncture site hemostasis, after which patients were mandated to strictly adhere to bed rest for a minimum of 20 h.

TRA-based TACE was also performed under local anesthesia with fluoroscopic guidance. After puncturing the left radial artery, a 0.035-inch hydrophilic wire (Terumo) was used to place a 4F MPA catheter (Cordis, FL, USA) in the celiac artery. Subsequent micro-catheterization and TACE procedures were then performed using the same approach as for TFA-based TACE procedures. When TACE was complete, mechanical compression with a radial compression device was used for radial puncture site hemostasis. Patients were directed to limit the movement of their left wrist for 2 h after the procedure.

All patients were permitted for free rest if there was no bleeding, hematoma, or pain at the puncture site.

### Assessment

TACE procedures were considered a technical success if successful femoral/radial artery puncture and tumor-supplying blood vessel catheterization were achieved. The time of TACE was calculated from the beginning of sterilization of the puncture site to the end of pressure of the puncture site. Procedure-related complications (Grades 1-5) were classified based on the Society of Cardiovascular & Interventional Radiology guidelines (15). TACE duration was the primary study endpoint. Secondary endpoints included the TACE technical success rate, fluoroscopy time, radiation dose, procedure-related complication, duration of postoperative bed rest, and duration of postoperative hospital stay.

### Statistical analysis

Skewed and normally distributed data were respectively compared using Mann-Whitney U tests and independent sample t-tests, whereas categorical data were compared with χ^2^/Fisher exact tests. SPSS 16.0 (SPSS, IL, USA) was used for all statistical analyses, with P < 0.05 as the threshold of significance.

### Ethics

This retrospective analysis has been approved by the Institutional Review Board of The Third People’s Hospital of Yancheng City, which determined that the requirement for written informed consent could be waived in light of the retrospective nature of this study.

## Results

### Patients

This study enrolled 83 HCC patients, of whom 41 and 42 respectively underwent TFA- and TRA-based TACE (**Table 1**). Both groups of patients exhibited similar baseline characteristics (**Table 1**).

**Table 1:** Patients’ baseline data.

	TFA (n = 41)	TRA (n = 42)	P value
Age (y)	61.1 ± 10.7	64.4 ± 9.8	0.142
Gender			0.944
Male	29	30	
Female	12	12	
BMI (kg/m^2^)	22.4 ± 2.8	23.5 ± 3.8	0.119
Etiology			0.391
HBV	33	32	
HCV	7	6	
Others	1	4	
BCLC stage			0.815
A	7	8	
B	34	34	
Child-Pugh class			0.752
A	30	32	
B	11	10	
INR			0.616
≥ 1.5	2	1	
< 1.5	39	41	
AFP (ng/ml)	323 (Q1: 18; Q3: 1919)	227 (Q1: 3; Q3: 6023)	0.176
Number of tumors			0.902
1	8	9	
2	4	3	
3 or more	29	30	
Diameter of the largest tumor (cm)	3 (Q1: 2; Q3: 5)	3.3 (Q1: 2.5; Q3: 4)	0.150
Number of TACE sessions	1.8 ± 0.9	1.6 ± 0.8	0.463

AFP, alpha-fetoprotein; BCLC, Barcelona Clinic Liver Cancer; BMI, body-mass index; HBV, hepatitis B virus; HCV, hepatitis C virus; INR, international normalized ratio; TACE, transarterial chemoembolization; TFA, transfemoral approach; TRA, transradial approach.

### TACE procedures

TACE-related outcomes for these patients are summarized in **Table 2**. The technical success rates in both groups were 100%, but the mean TACE duration in the TRA group was significantly lower than that in the TFA group (57.4 ± 23.5 min vs. 73.5 ± 23.3 min, P = 0.002). Comparable median fluoroscopy time (14 min vs. 13 min, P = 0.415) and radiation dose (159 Gy.cm^2^ vs. 160 Gy.cm^2^, P = 0.946) were observed in the TFA and TRA groups.

**Table 2:** Radiation dose and outcomes of the 2 groups.

	TFA (n = 41)	TRA (n = 42)	P value
Technical success rate	100%	100%	–
Entire TACE time (min)	73.5 ± 23.3	57.4 ± 23.5	0.002
Fluoroscopy time (min)	14 (Q1: 9; Q3: 23)	13 (Q1: 9; Q3: 18)	0.415
Radiation dose (Gy.cm^2^)	159 (Q1: 115; Q3: 204)	160 (Q1: 99; Q3: 231)	0.946
Access site hematoma	2 (4.9%)	2 (4.8%)	1.000
Postoperative bed rest			< 0.001
≥ 20 h	41	0	
< 20 h	0	42	
Postoperative hospital stay (d)	6.1 ± 3.4	7.0 ± 3.5	0.082

TACE, transarterial chemoembolization; TFA, transfemoral approach; TRA, transradial approach.

### Complications

Two patients in each group experienced Grade 2 puncture site hematomas (4.9% vs. 4.8%, P = 1.000, **Table 2**). All four of these hematomas were self-limiting and were managed through manual compression alone. No other complications were observed in either patient group.

### Hospitalization

All patients in the TFA group required ≥ 20 h of postoperative bed rest, whereas the same was not true for those patients in the TRA group (**Table 2**). Both groups exhibited a similar mean duration of postoperative hospitalization (6.1 ± 3.4 vs. 7.0 ± 3.5 days, P = 0.082).

## Discussion

In the present study, TRA- and TFA-based TACE procedures were compared with respect to their relative clinical efficacy, safety, and radiation dose in patients with inoperable HCC. A 100% technical success rate was achieved in both the TRA and TFA groups, consistent with the 95-100% success rates described previously for the TRA-based TACE treatment of HCC patients (14, 16). The high rate of technical success in patients undergoing TRA-based TACE in this study was primarily attributable to the use of 4F MPA catheters. MPA catheters have an angled tip (17), and their shape is compliant with the anatomy of the interface between the celiac artery and the proximal abdominal aorta.

While the fluoroscopy time in these two groups was similar, the overall operative duration was significantly shorter in the TRA group relative to the TFA group. This suggests that TACE procedures requiring fluoroscopic guidance were similar for both of these approaches, whereas the TRA technique is associated with similar perioperative procedures (e.g., preoperative puncture site sterilization, post-TACE application of pressure to the puncture site) as compared to the TFA technique. The similar fluoroscopy time in both groups accounts for the similar radiation doses for procedures performed using these two approaches.

Patients in the TFA and TRA groups also exhibited similar complication rates (4.9% and 4.8%, respectively), and these rates were consistent with rates reported previously when comparing TACE procedures in HCC patients performed using the TFA (5.5-7%) and TRA (1.9-2%) techniques (12, 14). All complications in this study were classified as Grade 2 events, in line with prior publications (12, 14).

Puncture site hematomas were the only reported complications for patients in this study. In other reports, radial artery occlusion has been found to impact 1.9-3.1% of patients undergoing TRA-based TACE, and this complication may be related to the repeat TRA punctures (8, 12, 14). But this complication was not observed in the present study. This discrepancy is likely attributable to the limited sample size of the present study and its retrospective design.

While the safety profile of the TRA technique was not superior to that of the TFA technique in this study, it was associated with a significantly reduced duration of postoperative bed rest. Patients were also able to move their legs on the bed after undergoing TRA-based TACE, significantly improving their overall procedural satisfaction. Li et al. (18) also found that TRA to hepatic arterial infusion chemotherapy (HAIC) is associated with greater improvement in the quality of life associated with the procedure compared with the TFA. The duration of postoperative hospitalization in both groups was similar in this study, as these patients underwent further treatments aimed at protecting the liver and gastric mucosa.

There are some limitations to this study. As it was a retrospective analysis, it faces a high potential for selection bias, although this risk is partially mitigated by the baseline data similarities in both groups. The power of the statistical analyses herein was also limited by the small sample size when evaluating endpoint outcomes, especially for some rare complications. Lastly, follow-up TACE-related data were unavailable for examination, although these outcomes were not the focus of this analysis.

## Conclusion

In summary, these results demonstrate that both TRA- and TFA-based approaches are suitable for TACE procedures in patients with inoperable HCC. Relative to the TFA, the TRA was associated with a significantly shorter arterial compression time and a marked reduction in the requirement for postoperative bed rest.

## Data Availability

The original contributions presented in the study are included in the article/supplementary material. Further inquiries can be directed to the corresponding author.

## References

[1] Huppert P. Transarterial chemoembolization of hepatocellular carcinoma. Radiologe. (2022) 62:225–33. doi: 10.1007/s00117-022-00972-1 35171312

[2] Titano J Noor A Kim E. Transarterial chemoembolization and radioembolization across barcelona clinic liver cancer stages. Semin Intervent Radiol. (2017) 34:109–15. doi: 10.1055/s-0037-1602709 PMC5453775 28579678

[3] Sangro B Salem R. Transarterial chemoembolization and radioembolization. Semin Liver Dis. (2014) 34:435–43. doi: 10.1055/s-0034-1394142 25369305

[4] Tsochatzis EA Fatourou E O’Beirne J Meyer T Burroughs AK. Transarterial chemoembolization and bland embolization for hepatocellular carcinoma. World J Gastroenterol. (2014) 20:3069–77. doi: 10.3748/wjg.v20.i12.3069 PMC3964379 24695579

[5] Zhou WJ Jin X Xu C Zhou XX Lv PH. Trans-radial versus trans-femoral approach for cerebral angiography: a meta-analysis of randomized controlled trials. Wideochir Inne Tech Maloinwazyjne. (2023) 18:235–43. doi: 10.5114/wiitm.2022.123309 PMC10481446 37680739

[6] Sakaoka A Souba J Rousselle SD Matsuda T Tellez A Hagiwara H. Different vascular responses to a bare nitinol stent in porcine femoral and femoropopliteal arteries. Toxicol Pathol. (2019) 47:408–17. doi: 10.1177/0192623318800726 30282527

[7] Posham R Biederman DM Patel RS Kim E Tabori NE Nowakowski FS. Transradial approach for noncoronary interventions: A single-center review of safety and feasibility in the first 1,500 cases. J Vasc Interv Radiol. (2016) 27:159–66. doi: 10.1016/j.jvir.2015.10.026 26706186

[8] Zhang X Luo Y Tsauo J Zhao H Gong T Li J. Transradial versus transfemoral access without closure device for transarterial chemoembolization in patients with hepatocellular carcinoma: a randomized trial. Eur Radiol. (2022) 32:6812–9. doi: 10.1007/s00330-022-09038-1 36018356

[9] Mortensen C Chung J Liu D Ho S Legiehn G Machan L. Prospective study on total fluoroscopic time in patients undergoing uterine artery embolization: comparing transradial and transfemoral approaches. Cardiovasc Intervent Radiol. (2019) 42:441–7. doi: 10.1007/s00270-018-2100-3 30374611

[10] Rao SV Cohen MG Kandzari DE Bertrand OF Gilchrist IC. The transradial approach to percutaneous coronary intervention: historical perspective, current concepts, and future directions. J Am Coll Cardiol. (2010) 55:2187–95. doi: 10.1016/j.jacc.2010.01.039 20466199

[11] Jolly SS Yusuf S Cairns J Niemelä K Xavier D Widimsky P. Radial versus femoral access for coronary angiography and intervention in patients with acute coronary syndromes (RIVAL): a randomised, parallel group, multicentre trial. Lancet. (2011) 377:1409–20. doi: 10.1016/S0140-6736(11)60404-2 21470671

[12] Du N Yang MJ Ma JQ Luo JJ Zhang ZH Yu TZ. Transradial access chemoembolization for hepatocellular carcinoma in comparation with transfemoral access. Transl Cancer Res. (2019) 8:1795–805. doi: 10.21037/tcr.2019.08.40 PMC8799211 35116930

[13] Karrowni W Vyas A Giacomino B Schweizer M Blevins A Girotra S. Radial versus femoral access for primary percutaneous interventions in ST-segment elevation myocardial infarction patients: a meta-analysis of randomized controlled trials. JACC Cardiovasc Interv. (2013) 6:814–23. doi: 10.1016/j.jcin.2013.04.010 23968700

[14] Hedjoudje M Barat M Dohan A Lucas A Dautry R Coriat R. Comparison between radial and femoral artery access for transarterial chemoembolisation in patients with hepatocellular carcinoma. Can Assoc Radiol J. (2024) 75:178–86. doi: 10.1177/08465371231186524 37563785

[15] Filippiadis DK Binkert C Pellerin O Hoffmann RT Krajina A Pereira PL. Cirse quality assurance document and standards for classification of complications: the cirse classification system. Cardiovasc Intervent Radiol. (2017) 40:1141–6. doi: 10.1007/s00270-017-1703-4 28584945

[16] Ghosh A Gupta V Al Khalifah A Akhter NM. Transradial versus transfemoral arterial access in DEB-TACE for hepatocellular carcinoma. J Clin Imaging Sci. (2022) 12:38. doi: 10.25259/JCIS_47_2022 36128344 PMC9479582

[17] Fu YF Wei N Zhang K Xu H. Subcarinal ventilation-assisted Y-shaped stent insertion under local anesthesia for patients with complex tracheobronchial stenosis: initial clinical experience. Diagn Interv Radiol. (2014) 20:330–4. doi: 10.5152/dir.2014.13498 PMC4463277 24989715

[18] Li B Li Q Peng L Xiang K Xu A. A randomized comparison of transradial and transfemoral approach in hepatic arterial infusion chemotherapy. Curr Med Imaging. (2023). doi: 10.2174/1573405620666230511094840. Epub ahead of print. 37165679

